# A novel approach to quantify different iron forms in *ex-vivo* human brain tissue

**DOI:** 10.1038/srep38916

**Published:** 2016-12-12

**Authors:** Pravin Kumar, Marjolein Bulk, Andrew Webb, Louise van der Weerd, Tjerk H. Oosterkamp, Martina Huber, Lucia Bossoni

**Affiliations:** 1 Huygens-Kamerlingh Onnes Laboratory, Leiden University, 2333 CA Leiden, The Netherlands; 2 Department of Radiology, Leiden University Medical Center, Leiden, The Netherlands; 3 Department of Human Genetics, Leiden University Medical Center, Leiden, The Netherlands

## Abstract

We propose a novel combination of methods to study the physical properties of ferric ions and iron-oxide nanoparticles in *post-mortem* human brain, based on the combination of Electron Paramagnetic Resonance (EPR) and SQUID magnetometry. By means of EPR, we derive the concentration of the low molecular weight iron pool, as well as the product of its electron spin relaxation times. Additionally, by SQUID magnetometry we identify iron mineralization products ascribable to a magnetite/maghemite phase and a ferrihydrite (ferritin) phase. We further derive the concentration of magnetite/maghemite and of ferritin nanoparticles. To test out the new combined methodology, we studied brain tissue of an Alzheimer’s patient and a healthy control. Finally, we estimate that the size of the magnetite/maghemite nanoparticles, whose magnetic moments are blocked at room temperature, exceeds 40–50 nm, which is not compatible with the ferritin protein, the core of which is typically 6–8 nm. We believe that this methodology could be beneficial in the study of neurodegenerative diseases such as Alzheimer’s Disease which are characterized by abnormal iron accumulation in the brain.

In many neurodegenerative diseases (ND), such as Alzheimer’s disease (AD), structural and molecular changes occur in the human brain which may lead to severe loss of memory and cognitive dysfunction. Increases (approximately 1 mM) of iron in specific brain regions of AD patients have been reported^1^, particularly associated with amyloid plaques which correlate with AD pathogenesis^2^. Despite the fact that a correlation between brain and iron dis-homeostasis has been suggested for many years^3^,^4^, a causal link between the two has not been proven so far, and some controversial results^5^,^6^,^7^ remain poorly addressed. Moreover, the chemical and magnetic properties of brain iron remain ill-defined. An overview of the iron ions and iron mineralization products potentially involved in the pathogenesis of ND are summarized in Table 1.

Using transmission electron microscopy (TEM) and superconducting quantum interference device (SQUID) magnetometry, magnetite nanocrystals in clumps of between 50 and 100 particles, isolated from the brain of four elderly healthy individuals and two AD patients^8^, were detected at a concentration of 3.95 ng/g (AD patients) and 4.2 ng/g (controls). This study was followed by SQUID magnetometry investigations of freeze-dried brain tissue of AD patients^9^ and brain tumor tissue from epileptic patients^10^. The work of Pankhurst and collaborators^9^ showed, for the first time, that superparamagnetic magnetite levels were higher in Alzheimer’s patients (0.75 *μg*/*g*) than in age-matched controls (0.12 *μg*/*g*). Among different iron-oxide compounds, magnetite deserves special attention because it contains the divalent iron, Fe(II), which is thought to catalyze the Fenton reaction, responsible for the production of the highly damaging hydroxyl radical and other radical species^11^,^12^,^13^,^14^,^15^. More recently it was proposed that Fe(II) may originate from ferritin’s inability to fully oxidize Fe(II) to Fe(III)^16^. This hypothesis has been supported by the observation that ferritin cores isolated from *ex-vivo* brain tissue of AD patients are two times more abundant in magnetite than in ferrihydrite, while the hexagonal ferrihydrite phase is dominant in age-matched control subjects^5^,^6^. However, these results appear quite controversial when compared to Nuclear Magnetic Resonance (NMR) measurements^17^, showing no significant difference between the spin-lattice relaxation rate of protons in ferritin samples purified from the brain of an AD patient and an age-matched control subject.

Ferritin is the main iron storage protein in the human body. It can store up to 4500 Fe(III) ions in its core, in a mineral nanocrystal named ferrihydrite^18^. Ferritin constituted the first experimental observation of a superantiferromagnet: its spin structure is made of an antiferromagnetic core, with an exchange field of 320 T, and a Nèel temperature in the range of 200–450 K^19^,^20^. Defect sites in the antiferromagnetic lattice, likely at the surface, give rise to uncompensated spins which can fluctuate about the easy axis, due to thermally activated processed (superparamagnetic behaviour). Below the critical temperature, the iron moments block parallel to the easy axis, thus giving rise to a non-zero magnetic moment in the range of 150–350 *μ*_*B*_.

Growing evidence of the role of iron in the development of neurodegeneration comes from recent *in-vitro* studies, reporting that amyloid aggregates are capable of accumulating Fe(III) and reducing it to the ferrous state Fe(II)^21^,^22^. In addition, studies on clustered neuronal networks showed that the addition of amyloid and magnetite nanoparticles to the cell culture induces the loss of neuronal activity and degrades the functional organization and connectivity of neuronal networks^23^. Therefore, the need for an analytic study of different iron-oxide compounds in the brain of patients with neurodegenerative diseases is more compelling than ever.

While techniques such as atomic absorption spectrometry and inductively coupled mass spectrometry can quantify metals in selected brain regions, they lack the specificity to measure their oxidation state and magnetic properties. Magnetic resonance imaging (MRI), weighted by 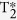 24,25,26, can be used to indirectly image iron *in-vivo* and *ex-vivo*, but the data interpretation remains much debated. Such measures are indirect and affected by artifacts from magnetic field inhomogeneities, due to tissue-tissue interfaces, air-tissue interfaces and myelin^27^,^28^,^29^,^30^. In addition, amyloid accumulation itself may increase the transverse relaxation rate^31^, even after treatment with an iron chelator, thus suggesting that the relaxation time is likely not affected by the chelatable iron ions^32^. Finally, the use of histology, often combined with MRI^31^,^33^,^34^, is non-quantitative and its reproducibility is very much dependent on the details of sample handling^35^.

In this manuscript, we introduce a novel approach, by complementing SQUID magnetometry with Electron Paramagnetic Resonance (EPR), to study the properties of different iron forms in the human brain. To illustrate the method with a practical example, we studied formalin fixed human brain tissue, which was sectioned from the temporal cortex of an AD patient and a healthy gender-matched control (HC). Middle temporal gyrus was chosen since MRI 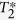-weighted data suggest increased iron levels in this anatomic region^36^. As a comparison and reference, we studied a sample of commercial horse spleen ferritin and a Fe(III)-EDTA solution.

The effects of formalin fixation, together with airborne and cauterization effects on iron mineralization products in the brain, have been studied by Dobson *et al*.^37^. Their results suggest that after only one week in formalin, the tissue magnetization is reduced, while the coercivity spectrum remains similar. This effect is suggested to originate from formation of formic acid, which could affect the magnetic phase. In our study, only formalin fixed tissue has been used, and therefore one should recognize that the absolute magnetite/maghemite phase may be underestimated. Frozen tissue (when available) should always be preferred. However, it may still be reasonable to compare variations of magnetite fractions between samples stored in formalin for a comparable period of time.

Using 9 GHz EPR, we measured the concentration of iron ions of the AD and HC samples. Using SQUID magnetometry, we quantified magnetic iron-oxide nanoparticles, in the same patients. Our Isothermal Remanent Magnetization (IRM) data show that a magnetite/maghemite phase is present in the tissue, confirming previous results^9^. Furthermore, from the IRM data at different temperatures, we quantified the concentration of both magnetite/maghemite and ferritin nanoparticles, and their size distribution.

The combination of these techniques offers quantitative information about specific iron forms in the human brain, which is relevant to the study of neurodegenerative diseases, both in terms of the biochemistry of the disease, and to improve current diagnostic tools. In particular, we provide concentrations of different iron forms without the need of processing the tissue. We foresee that such an approach will be beneficial in complementing *ex-vivo* MRI studies.

Although our study presents SQUID and EPR data obtained from the brain tissue of an AD patient and a healthy control, the primary goal of our manuscript goes beyond the simple comparison between the two subjects. In this manuscript, we aim at introducing a new methodology which will help to unravel the complex problem of iron overload in neurodegenerative diseases, especially when combined with the associated, and commonly used, MRI technique.

## Results

### EPR results

**Figure 1:**
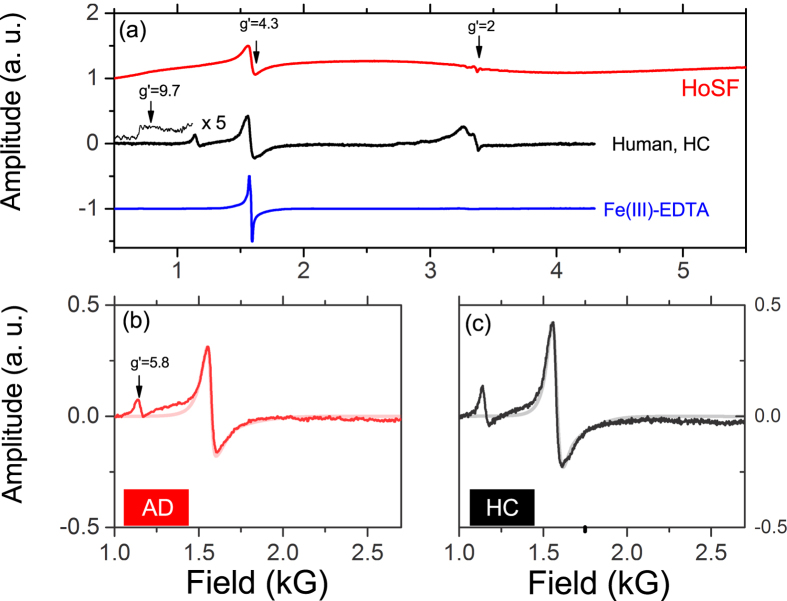
EPR spectra of the different samples at 12 K and spectra simulation. (**a**) EPR spectrum acquired at 12 K of Fe(III)-EDTA reference sample (blue curve), human sample of a healthy individual (black curve), and horse spleen ferritin (HoSF) (red curve). The human spectrum shows two main bands: one typical of the iron high spin state, at g′ = 4.3, and the other typical of copper, at g′ = 2. The data are compared with the HoSF spectrum acquired at 170 K, above the blocking temperature of ferritin, and the Fe(III)-EDTA reference. The spectra intensities are re-scaled. EPR spectra and simulation for the two brain tissues are shown: AD (**b**) and healthy control (HC) (**c**). The arrow indicates the weak g′ = 5.8 band at low fields. Raw data are in black/red, while the simulated spectra are in grey/pink.

Continuous-wave 9 GHz EPR spectra were acquired on human brain tissue, at 12 K. Figure 1(a) shows a representative spectrum of human HC together with the reference Fe(III)-EDTA and a commercial lyophilized horse Spleen Ferritin (HoSF) sample. In all samples, the same characteristic iron, high spin state, g′ = 4.3 band is observed. The spectrum of HoSF, measured at 170 K, displays also a broad band centered around g′ = 2, due to iron bound to the superparamagnetic ferrihydrite mineral^38^,^39^,^40^. This signal disappears around 20–30 K (data not shown), where the transition to the blocked state occurs^41^,^42^. In addition, the human tissue displays a weak band at g′ = 9.7 and a structured signal around g′ = 2, mostly due to copper, and radicals. Figure 1(b,c) show the g′ = 4.3 iron signal of the AD and HC human samples, respectively. Here a weak band around g = 5.8, likely due to methemoglobin^43^, is observable.

To thoroughly characterize our EPR iron spectrum, we carried our a progressive power saturation experiment, from which we derived the product of the relaxation times for the g′ = 4.3 band, at 12 K (see Supplementary Information).

In order to understand the biological environment of this iron signal, it is necessary to know the Hamiltonian parameters. The high-spin ferric state (S = 5/2, *d*^5^) is described by the spin Hamiltonian:


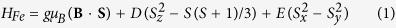


where *g* is the Landé factor, *μ*_*B*_ the Bohr magneton, *B* is the applied field and *S* the spin operator. The two final terms represent the zero-field splittings, where D is the axial splitting, and E the rhombic splitting. These originate from electrostatic interaction among multiple unpaired electrons in the ions, typical of high spin Fe(III)^43^. The traceless *D* tensor can be written in the principal axes system as:


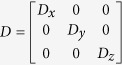


with *D* = 3/2*D*_*z*_, and *E* = (*D*_*x*_ − *D*_*y*_)/2. The 9 GHz spectrum is, for the most part, determined by the ratio between D and E, which in turn reflects the deviation of the crystal from ideal tetrahedral symmetry (D = E = 0). In the common case for non-heme iron proteins, *λ* = *E*/*D* = 1/3, and at 9 GHz, the microwave energy is smaller than D (*hν* < *D*). Therefore the six states ±1/2, ±3/2, ±5/2 become mixed, thus allowing forbidden transitions. In this specific case, the ±3/2 transition becomes more intense due to its isotropic character, whereas weaker features are observed at 700 G (g′ = 9.7), arising from the ±1/2 doublets^43^,^44^. We report the simulated Hamiltonian parameters in Table 2. These parameters are typical of most of the non-heme iron, in an octahedrally distorted ligand field, in proteins. A discussion of the Cu signal can be found in the Supplementary Information.

Finally, the simulated curve can be used to derive the concentration of the Fe(III) spins in the tissue, by using the following expression:


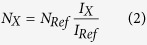


where *N*_*X*_ is the number of iron spins in the tissue, *N*_*Ref*_ is the number of iron spins in the reference sample, which is equal to 10 nmol in our case, *I*_*X*_ is the second integral of the human tissue spectrum, and *I*_*Ref*_ is the second integral of the reference. By scaling for the tissue mass, we derive the iron concentration. In this study, the HC sample contains 1.9 ± 0.4 *μg*/*g* and the AD sample contains 2.1 ± 0.4 *μg*/*g* iron ions. The error takes into account the uncertainty in the tissue mass, together with the simulation error.

### SQUID results

**Figure 2:**
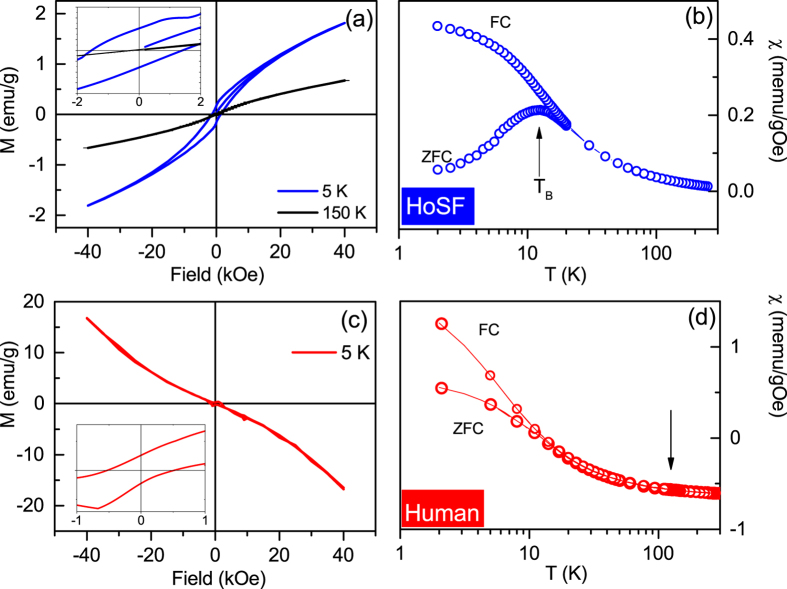
SQUID study of HoSF and a human (AD) brain tissue: comparison between different methods. (**a**) Hysteresis measurements of a sample for HoSF at 5 K (blue line) reached in ZFC conditions, and the closed hysteresis at high temperature, 150 K (black line) indicating the superparamagnetic behaviour. The inset is an expansion of the low field region. (**b**) Static spin susceptibility of the same sample measured in ZFC and FC conditions (100 G). The arrow marks the DC blocking temperature. (**c**) Hysteresis measurements of a sample of freeze dried temporal cortex of the AD patient. The inset is an expansion of the low field region, after correcting for the diamagnetic contribution. (**d**) Static spin susceptibility of the human tissue at 100 G in ZFC and FC conditions. Here the arrow indicates the temperature at which the Verwey transition would be expected for bulk magnetite (see text).

Zero-Field Cooled (ZFC) and Field Cooled (FC) magnetization curves at low field (100 G) and the hysteresis curves at different temperatures were obtained (Fig. 2) for lyophilized human brain tissue as well as a standard sample of 37 mg of freeze-dried HoSF. The hysteresis curves of HoSF (Fig. 2(a)) are in good agreement with literature^45^, showing a rather high coercitivity phase with *H*_*c*_ = 1400 Oe, at 5 K, while *H*_*c*_ = 0 at 150 K, in agreement with the superparamagnetic behaviour expected for ferritin nanoparticles. The ZFC/FC study shows a peak at 12.33 ± 1 K, marking the DC blocking temperature (Fig. 2(b)). ZFC and FC magnetization curves at low field and the hysteresis curve at 5 K were obtained also for the human sample. Here the magnetization *M* is dominated by a diamagnetic signal (Fig. 2(c)). Similarly to the pure ferritin sample, the hysteresis loop opens at low temperature, therefore indicating the presence of blocked magnetic particles, with a low coercivity of *H*_*c*_ = 620 ± 10 Oe. Additionally, the low field susceptibility *χ* shows a bifurcation of the ZFC/FC curves around 15 K (Fig. 2(d)), but no defined peak is observed, possibly because the signal overlaps with other paramagnetic species. It is worth noting that no peak at 125 K was observed, in the ZFC/FC curves. Such a peak is reported in bulk magnetite, as well as in frozen brain tissues^10^,^46^, as result of a charge order Verwey transition. The lack of a Verwey transition here does not necessarily imply the absence of a magnetite phase in the sample. Indeed, the ability to observe this transition depends on several conditions such as particle size^47^, particle spatial arrangement, i.e. whether they are isolated or forming chains/clusters^48^, and particle structural changes. When magnetite is in the form of nanoparticles, it becomes very sensitive to oxidation by oxygen, which might result in stoichiometric changes leading to the disappearance of the transition^49^.

**Figure 3:**
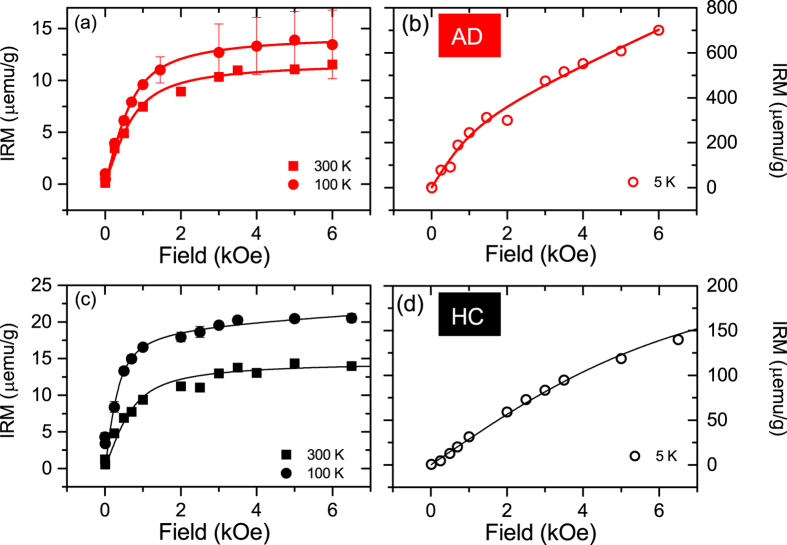
IRM SQUID study of freeze-dried human brain tissue, from the AD patient and the HC. (**a**) Isothermal Remanent Magnetization (IRM) of the AD sample, measured at 300, 100 K and 5 K (**b**). (**c**) Isothermal Remanent Magnetization (IRM) of healthy control sample, measured at 300, 100 K and 5 K (**d**). Error bars (indicated only for the 100 K data sets) are experimental errors due to the fitting of the raw voltage induced in the SQUID pick-up coil. Solid lines are empirical fits to the Langevin curve.

In addition to hysteresis and ZFC/FC curves, we also measured the Isothermal Remanent Magnetization (IRM) of the human brain tissues (Fig. 3) at 300 K, 100 K and 5 K.

The high temperature curves saturate around 3000 Oe (Fig. 3(a,c)), which is a value expected for magnetite/maghemite^8^. From the IRM saturation value we can determine the concentration of the magnetite/maghemite phase. The IRM of the AD patient displays a saturating magnetization of *M*_*s*_ = 11.8 ± 2.5 *μ*emu/g at 300 K, and 14.5 ± 2.5 *μ*emu/g at 100 K. When we compare these values with the saturation magnetization of bulk maghemite, i.e. 69–84 emu/g^47^,^50^,^51^, we derive a concentration of 155 ± 33 ng/g, and 189.5 ± 32.6 ng/g, at room temperature and at 100 K, respectively. The HC sample indicates a concentration of 188 ng/g (at 300 K) and 277 ng/g (at 100 K). The spread of *M*_*s*_ for pure maghemite depends on the particle size distribution. Additionally, if magnetite is considered, *M*_*s*_ ranges between 71 emu/g for nanoparticles of 30–49 nm^47^ to 92 emu/g for bulk magnetite. Therefore, on the sole basis of *M*_*s*_, maghemite and magnetite are hardly distinguishable. Indeed, the identification of magnetite (Fe(II)Fe(III)_2_O_4_) and maghemite (*γ*-Fe(III)_2_O_3_) is quite intricate, because both phases possess the same spinel structure and almost identical lattice parameters^52^. Since it cannot be excluded that a polycrystalline multi-phase structure is present in these nanoparticles, we always refer to the magnetite/maghemite phase, in this manuscript.

The IRM curve at 5 K does not saturate in the field range under study (Fig. 3(b,d)), which is an indication of ferrihydrite, the mineral core of ferritin. Indeed, ferritin behaves like a superantiferromagnet below the Néel temperature of ~500 K^19^, and it displays a non-zero magnetic moment due to two contributions: (i) uncompensated magnetic moments due to defects in the AF lattice, and (ii) slight canting of the AF sublattices, giving rise to a linear term in field *χ*_*AF*_*H*^19^. From the blocking temperature, measured by the SQUID on the HoSF sample, we deduced an energy barrier of about *E*_*a*_ ~ 26 meV, in good agreement with the anisotropy barrier of single ferritin molecules estimated by Nolte *et al*.^53^. By using the same *E*_*a*_, we calculated a critical blocked diameter at 5 K, of ~6 nm^50^. This result suggests that most of the ferritin cores are blocked at this temperature, thus dominating the SQUID signal. It is worth noting that the remanent magnetization at these small particle sizes is likely to include a small contribution from frustrated surface spins.

If we now compare the magnetic moment measured at 6 kG and 5 K (Fig. 3(b,d)), with the known value of a pure sample of ferritin, we obtained a ferritin concentration of 1.4 mg/g (dry weight), or alternatively ~210 ± 21 *μg*/g (wet weight) for the AD sample, and ~40 ± 4 *μg*/g (wet weight) for the HC, after correcting for the contribution of the blocked magnetite nanoparticles. We summarize the results of this work in Table 3.

Finally, we carried out a time dependent study of the thermoremanent magnetization at 5 K, in order to derive the ferritin particle size distribution, which is presented in the next section.

## Discussion

We begin our discussion with the results of the EPR investigation. EPR is sensitive to paramagnetic centers and in the context of the present study iron ions are of particular interest, here the Fe(III) ions (see Tables 1 and 2). As a 3*d*^5^-ion, Fe(III) can be in the low-spin state, S = 1/2 (not observed here), or in the high-spin state in which all 5*d* electrons are unpaired, generating an S = 5/2 spin state. The position of an EPR resonance line is given by the effective g value (g'), which defines the magnetic field strength-microwave frequency combination at which the signal is observed. These g-values are characteristic for the iron-ion electronic state and therefore diagnostic of the type of Fe(III). In addition to the resonance position, the shape of the signals and their width, defined by the peak-to-peak linewidth (*G*_*pp*_), is also characteristic. As these EPR signals are overall broad, they are often referred to as *bands*. The quantity of the species contributing to each signal is determined by integration of the signal in question as described in the Supplementary Information.

Metal ions in different *ex-vivo* human brain tissues have already been characterized by EPR^54^,^55^,^56^. Given the spectra we observed and the analysis of their Fe(III) signals, there are various iron-bound proteins in the human brain that can contribute to the signals. However, we can restrict our search to a limited number. Indeed, the g′ = 5.8 band, commonly associated with Fe(III)- heme iron in methemoglobin, suggests that the residual blood^57^ in the tissue is a minor fraction and it can be neglected in the spectral simulation, due to its different g′ value. Transferrin, a possible candidate for the band observed here^40^ has a typical linewidth of ~125 G_*pp*_ at 12 K and a splitting of the g′ = 4.3 peak of ~32 G^43^,^44^,^57^. In contrast, the g′ = 4.3 band observed has a width of 57 ± 2 G_*pp*_ across all temperatures, and no splitting. Conversely, iron bound to low molecular weight complexes, also called “loosely bound iron”^39^,^58^,^59^,^60^, presents spectral features similar to our signal (Fig. 1). Indeed, Moser *et al*. observed an increase in the intensity of g′ = 4.3 signal in a tissue homogenate and in a ferritin solution upon treatment with ascorbate and deferrioxamine. We therefore propose that the observed iron signal is likely mononuclear high-spin iron in sites of low symmetry, possibly bound to low molecular weight complexes^58^. Weakly or loosely bound iron can be used in catalytic cycles to produce the very damaging hydroxyl radical, via Haber-Weiss and Fenton reactions^11^,^58^,^61^. Reagents of these reactions are the hydrogen peroxide and superoxide radical, which are both produced by mitochondria. Weak iron chelation in the presence of an antioxidant agent such as ascorbate (vitamin C) can promote the production of the hydroxyl radicals. Loosely bound iron should be less than 5% of the total iron within cells^59^. Total iron levels in healthy aged subjects vary from 122 *μg*/*g*^62^ (middle temporal gyrus) to 28 *μg*/*g*^27^ (temporal cortex): therefore we would expect to measure iron concentrations in the range of 6.1–1.4 *μg*/*g*. Our method derives values of 1.9 ± 0.4 *μg*/*g* and 2.1 ± 0.4 *μg*/*g* Fe(III) ions for the HC and AD patient, respectively, in agreement with the expectations.

Due to its high sensitivity, SQUID magnetometry is an ideal tool to characterize the iron mineralization products in the brain, through, their magnetic properties. Typically, SQUID measures the total magnetic moment of every spin-carrying component of the sample. For monophasic systems, the study of the hysteresis curve can provide an accurate description of the sample. However, in a complex system such as human tissue, modeling all the sample fractions is much more complicated. For the purpose of the present study, measurements sensitive only to the permanent magnetic iron forms are preferable, as this type of iron is complementary to the iron species measured by EPR, and it is the one that can substantially affect the nuclear spin relaxation times^63^. Therefore we chose the IRM method, by which the sample is brought to some initial state of magnetization M_*i*_ by an applied field, and then the field is turned off very fast. At high temperatures, the particles that are in the superparamagnetic state, such as ferritin, will quickly reverse their magnetization. On the other hand, the blocked particles will contribute to a saturating magnetization^64^. By comparing the saturation magnetization *M*_*s*_, of the human tissue, with the respective pure compounds, we can derive the concentration of the blocked particles. Within the tissue of AD patient, we obtained a concentration of maghemite particles of 155 ± 33 ng/g, and 189.5 ± 32.6 ng/g, at room temperature and at 100 K, respectively. The HC sample indicates a concentration of 188 ng/g (at 300 K) and 277 ng/g (at 100 K). When these values are rescaled for the wet tissue mass and compared with the results of other authors, we find agreement with Hautot *et al*.^46^, where magnetite levels have been derived by the IRM curve of freeze-dried tissue of neuroferritinopathy patients and controls, while less agreement is observed with the work of Pankhurst *et al*.^9^ where magnetite levels reached up to ~1 *μg*/*g* in the temporal cortex of female AD patients. We notice that the latter work reports levels of magnetite nanoparticles smaller than 20 nm, whereas our study focuses on larger magnetite particles which are already blocked at room temperature.

**Figure 4:**
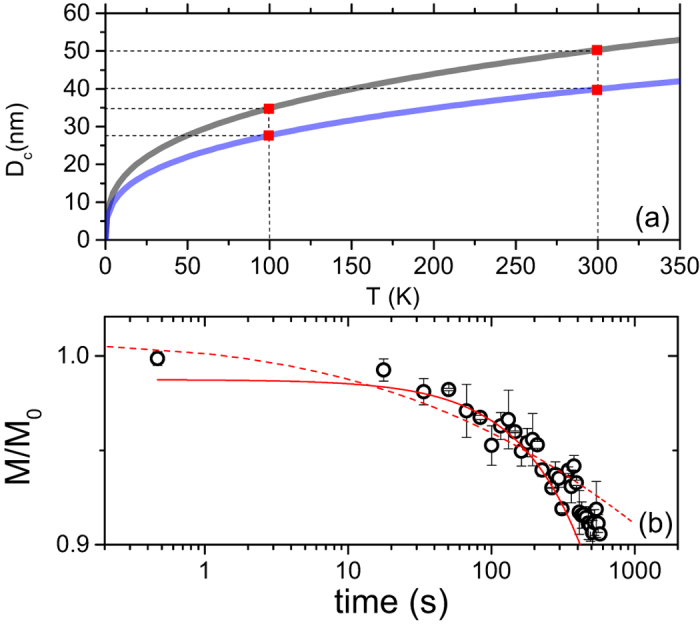
Illustration of the maghemite, magnetite and ferritin particles size, as derived by the Néel model, and the thermoremanent magnetization. (**a**) Simulation of the critical particle size (*D*_*c*_), for maghemite (black curve) and magnetite (blue curve), as a function of the temperature. The red points refer to the measured temperatures. **(b)** Decay of the magnetization as a function of the time, after turning off a field of 500 Oe. Data are acquired at 5 K, i.e. below *T*_*B*_ of ferritin. Data have been normalized. The dashed solid line is the simulated curve described by a log-normal particle distribution, while the red solid curve is the fit to the single particle relaxation.

Considering the anisotropy constant *K* = 2.6 × 10^5^ erg/cm^3^ for maghemite and almost a factor of two smaller for magnetite^65^,^66^, and assuming a cubic particle shape^8^ of volume V, we can derive the minimal volume of the blocked particles. We equate the measurement time, on the order of 10^2^ s, to the Néel relaxation time^50^:


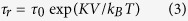


where T is the temperature and *k*_*B*_ is the Boltzmann constant. Figure 4(a) shows the critical diameter for magnetite/maghemite particles, as a function of the temperature, under the assumption of a unique particle size. At 300 K it is found that all particles larger than ~40–50 nm can give rise to a blocked magnetic moment, while at 100 K smaller particles, namely the ones above 27–35 nm, can be blocked. The spread of values is due to the uncertainty on the exact crystal phase. Given the size of these particles, we exclude the possibility that they are found within the core of ferritin, which is approximately 6–8 nm, although they could still originate from aggregated ferritin. We note that the Vogel-Fulcher law has been excluded from the analysis because of the small inter-particle interaction. Indeed if we calculate the Vogel-Fulcher temperature as a rough estimate of the temperature related to the dipolar interaction strength between two nanoparticles^67^, we derive a negligible Vogel-Fulcher temperature.

From the IRM curves measured at 5 K, we derive a ferritin concentration of ~210 ± 21 *μg*/g and ~40 ± 4 *μg*/g (wet weight), for the AD and HC patients, respectively. The AD value is almost six times larger than estimated by Dexter *et al*.^68^ using the whole cerebral cortex, while it is two times smaller than the results of Connor *et al*. for the frontal cortex^69^. On the other hand, the HC data agree well with literature. As ferritin concentration varies among individuals and among different anatomical regions of the brain, such differences are not surprising.

Further information about the ferritin size distribution can be derived from the thermoremanent magnetization, which implies measuring *M*, as a function of the time, after turning off the magnetic field. In the case of a unique particle size the IRM will decay exponentially, *M*_*r*_ = *M*_0_ exp(−*t*/*τ*_*r*_)^50^. However, for an assembly of particles, the magnetization has to be weighted over the volume size distribution *f*(*V*)^64^:


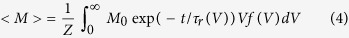


where:


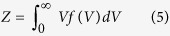


If we assume a uniaxial anisotropic constant of *K*/*k*_*B*_ = 0.825 K/nm^3 ^53, we can qualitatively reproduce the thermoremanent magnetization at 5 K (Fig. 4(b)), by employing a log-normal volume distribution for ferritin nanoparticle volumes with median radius equal to 3.9 nm and standard deviation equal to 2 nm. Even if a particle size distribution would be expected in a human tissue, the quality of the data in Fig. 4(b) does not allow one to prefer one model over the other.

It is worth noting that magnetometry measurements do not provide information on the location and spatial distribution of magnetite/maghemite throughout the sample, whereas MRI 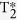-weighted images could potentially address this question. However, these measurements are not sensitive enough to detect the low concentrations of biogenic nanoparticles found here using 1.5 T clinical scanners^70^. Therefore, high field MRI scanners should be employed.

Finally, throughout this study formalin fixed tissue was the starting material. Even if the possibility of metal leaching out of the tissue, due to formalin fixation, cannot be excluded^71^, formalin fixed tissue was the choice due to the following considerations: (i) fresh-frozen tissues are rarely available so fixed tissue is often the chosen material; (ii) the methodology requires minimal sample preparation (See Materials and Methods); (iii) formalin fixed tissue contains only a minimal amount of blood, thus making the EPR spectra simulation more straightforward; (iv) our methodology allows a direct comparison with MRI results on the same tissue. A systematic comparison of MRI (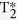-weighted) with EPR and SQUID on several human brain samples will be the subject of a follow-up study.

## Conclusions

Summarizing, we have shown that the combination of EPR and SQUID magnetometry offers unique insights into the study of the iron accumulation problem in the human brain. To the best of our knowledge, this is the first time that these two techniques have been used together to study brain magnetism.

We studied a few mg of tissue sectioned from the temporal cortex of an AD patient and a healthy gender-matched individual. By means of EPR, we selectively targeted low symmetry paramagnetic iron. We derived concentrations in the order of few *μ*g/g (w/w), which is in good agreement with the low molecular weight iron pool in the temporal cortex. Since EPR is selective only for the paramagnetic iron, we complemented our study with SQUID magnetometry which, conversely, is able to detect diluted magnetic iron nanoparticles via the IRM method. Using SQUID magnetometry we derived magnetite/maghemite concentrations of the order of hundreds of ng/g and ferritin concentrations of hundreds of *μ*g/g, in reasonable agreement with literature data. The concentrations of magnetite/maghemite and ferritin observed by SQUID are too low to be detected by EPR, thus showing the advantages of using both methods. The main advantage of our methodology lays in its sensitivity and in the ability of providing concentrations of different mineralization products of iron, without the need of processing the tissue, and/or isolating the proteins.

## Materials and Methods

### EPR sample preparation

Formalin-fixed human brain tissues of an AD patient (89 yr, female, Braak Stage: 4 C) and a healthy control (72 yr, female) were obtained from the Netherlands Brain Bank (NBB) of Amsterdam, after receiving informed consent. Brain tissue of a 70 yr old female control subject was used for the progressive power saturation experiment described in the Supplementary Information. Human brain tissue of AD patient or control as confirmed by neuropathological examination in agreement with the guidelines of the ethics committee of the LUMC. Patient anonymity was strictly maintained. All tissue samples were handled in a coded fashion, according to Dutch national ethical guidelines (Code for Proper Secondary Use of Human Tissue, Dutch Federation of Medical Scientific Societies).

Middle temporal gyrus of Alzheimer’s and control brains were dissected using ceramic scalpels and handled with non-metallic tweezers to prevent metal contamination of the sample. In order to protect the tissue from being damaged by water crystallization and therefore affecting the crystal field symmetry, we followed the protocol outlined below^72^. In short, the tissues were stored in a solution of 4% Paraformaldehyde (PFA) 0.1 M Phosphate Buffer (PB) 10.5% sucrose, for at least 4 hours. The tissues were then immersed into a solution of 4% PFA 0.1 M PB, 30% sucrose, overnight. This protocol ensures the elimination of water from the cells, and partially washes out the remaining blood. Afterwards, the tissues were wiped from remaining solution, weighted, and put into a Suprasil 4 mm tube and then plunged into liquid nitrogen. A piece of tissue of few tens of mg was enough to obtain the signal described above. The AD sample weights 19 mg, while the HC samples weight 78 and 60 mg. In order to determine the absolute iron spin count, we used a standard solution of 125 *μL* of Fe(III)-EDTA, prepared with glycerol/water, with a final iron spin concentration of 80 *μ*M. Spectra of the empty cavity, the empty Suprasil tube and tube containing 0.1 mL of the final sucrose solution were also acquired.

HoSF was purchased from Sigma-Aldrich (Sigma F4503).

### EPR method

The 9 GHz continuous wave (cw) EPR measurements were performed at cryogenic temperatures using an ELEXSYS E680 spectrometer (Bruker, Rheinstetten, Germany) equipped with a rectangular cavity. The microwave frequency was 9.4859 GHz, modulation frequency 100 kHz, power attenuation 20 dB, receiver gain 60 dB and modulation amplitude 6 G_*pp*_. The accumulation time was 20 min per spectrum. In order to check the stability of the experimental setup, which is particularly critical for quantitative EPR analysis, the Fe(III)-EDTA reference was measured at the beginning and at the end of each day of measurement. Each sample was placed into a helium flow cryostat. For the quantitative analysis, first derivatives of the EPR spectra were acquired at 12 K. This temperature was chosen to achieve (i) a good signal-to-noise ratio, (ii) relatively short relaxation times, in order to prevent saturation, and (iii) to suppress the broad signal of superparamagnetic Fe^41^.

### SQUID magnetometry sample preparation

Formalin-fixed human brain tissue was dissected using ceramic scalpels and handled with non-metallic tweezers. Tissues of about 1 cm^3^ were plunged into liquid Nitrogen and then freeze-dried over a period of minimum 48 h, so that a pellet was obtained. We observed a mass loss of about 85%, after freeze drying. The pelleted sample was pressed into a gel capsule with the help of a cotton swab. The gel capsule was then inserted into the plastic straw, used as sample holder, which was loaded into the SQUID magnetometer, equipped with an RSO (reciprocating sample operation) unit. Given the small concentration of ferritin and magnetite/maghemite in the sample, the use of the RSO unit, sensitive to 10^−8^ emu (10^−11^ Am^2^), was necessary.

### SQUID magnetometry method

As discussed in the Results section, in addition to the more conventional method of ZFC/FC and hysteresis, we decided to specifically target the magnetically diluted particles by measuring the Isothermal Remanent Magnetization (IRM). We did this by turning on the magnetic field (in no-overshoot mode), waiting for a few seconds, and then quenching the magnetic field, in the same mode. We expect that the para/dia-magnetic host will not contribute to the measured longitudinal magnetic moment *μ*. Conversely, in presence of a spontaneous magnetization, *μ* will be non-zero. We then iterated the measurement by progressively increasing the initial value of the magnetic field. IRM curves were performed at three different temperatures: 300 K, 100 K and 5 K. The lower temperatures were reached in ZFC mode after demagnetizing the sample. Is is important to note that the initial state of the FC magnetization is dependent on the thermal history of the sample. Therefore, we always worked in ZFC condition, thus making sure that the initial thermal magnetization *M*_*i*_ was zero, within experimental error. Moreover, given the low intensity of the signal, it is crucial to have an accurate zero field value (±1 G). This was calibrated with a paramagnetic Pd sample. Additionally, in order to prevent spurious fields in the magnet coils, the magnet reset option was activated before measuring every new sample. A drawback of this method is the large helium boil off, during the magnet reset: the daily helium consumption amounted to about 15% per day.

### LA-ICP-MS method

Formalin fixed tissue (wet weight ~50 mg) was, after washing with ultrapure water, destroyed using 0.3 ml HNO_3_ at 90 °C for 2 hours. To ensure complete tissue destruction, 100 *μL* H_2_O_2_ was added to the tissue solution and heated for 1 hour at 90 °C. Tubes were filled till 10 mL and iron concentrations were measured with LA-ICP-MS.

## Additional Information

**How to cite this article**: Kumar, P. *et al*. A novel approach to quantify different iron forms in *ex-vivo* human brain tissue. *Sci. Rep.* **6**, 38916; doi: 10.1038/srep38916 (2016).

**Publisher's note:** Springer Nature remains neutral with regard to jurisdictional claims in published maps and institutional affiliations.

## Supplementary Material

Supplementary Information

## Figures and Tables

**Table 1:** Overview of the possible forms of iron present in the human brain.

	Name	Toxicity
**Weakly bound iron ions**		
Fe(II)	ferrous iron	catalyzes ^•^OH production via Fenton reaction
Fe(III)	ferric iron	possibly converted into Fe(II) by amyloid and/or Haber Weiss reaction
Fe	total amount of iron in the tissue	
**Iron oxide possibly outside Ft**		
Fe_3_O_4_/*γ* − Fe_2_O_3_	magnetite/maghemite	carrier of Fe(II)/oxidation product of magnetite. It affects neuronal cell cultures together with amyloid
Fe_2_O_3_ • 0.5 H_2_O	ferrihydrite	—
**Iron oxide possibly outside Ft**		
Fe_3_O_4_/*γ* − Fe_2_O_3_	magnetite/maghemite	carrier of Fe(II)/oxidation product of magnetite. It affects neuronal cell cultures together with amyloid. It may originate from “pathological Ft”

Their suggested toxicity is also shown. ‘Ft’ refers to ‘ferritin’.

**Table 2:** Hamiltonian parameters of the HC human brain sample (the spectrum of the AD patient can be simulated with similar parameters).

*Fe*	D (GHz)	|E/D|	g_x_	g_y_	g_z_
	20.96	0.3324	1.83	1.998	2.0151

As the iron spectrum is not very sensitive to changes in the parameters, we report a spread of possible values in the Supplementary Information.

**Table 3:** Summary of the results presented in this manuscript.

	Results AD sample	Results HC sample
**Weakly bound iron ions**		
Fe(III)	2.1 ± 0.4 *μg*/*g*	1.9 ± 0.4 *μg*/*g*
Fe	218 *μg*/*g*	—
**Iron oxide possibly inside Ft**		
Fe_2_O_3_ • 0.5 H_2_O	210 ± 21 *μg*/*g*	40 ± 4 *μg*/*g*
**Iron oxide possibly outside Ft**		
	mmc at 300 K	mmc at 300 K
Fe_3_O_4_/*γ* − Fe_2_O_3_	155 ± 33 ng/g	188 ng/g

Total Fe concentration was measured on the AD sample, by Laser Ablation Inductively Coupled Plasma Mass Spectrometry (see Materials and Methods). ‘mmc’ stands for magnetite/maghemite concentration. The values refer to the room temperature measure.
