# Supplementary material for: A novel approach to quantify different iron forms in ex-vivo human brain tissue
Source: Sci Rep. 2016 Dec 12;6:38916. doi: 10.1038/srep38916 (PMC5150947; doi:10.1038/srep38916)
Supplement: Supplementary Information [file srep38916-s1.pdf]

SUPPLEMENTARY INFORMATION

A novel approach to quantify different iron forms in *ex-vivo* human brain tissue

Pravin Kumar,[1] Marjolein Bulk[2,3], Andrew Webb,[2] Louise van der Weerd,[2,3] Tjerk H. Oosterkamp,[1] Martina Huber,[1] and Lucia Bossoni[1a]

[1] *Huygens-Kamerlingh Onnes Laboratory, Leiden University, 2333 CA Leiden, The Netherlands*

[2] *Department of Radiology, Leiden University Medical Center, Leiden, The Netherlands and*

[3] *Department of Human Genetics, Leiden University Medical Center, Leiden, The Netherlands*

[a] corresponding author: L. Bossoni, bossoni@physics.leidenuniv.nl

PROGRESSIVE POWER SATURATION EXPERIMENT

An estimate of the relaxation times of the iron signal in the human samples can be obtained by performing a progressive power saturation experiment. As derived by Portis [1], it is convenient to plot the signal amplitude normalized by the square root of the incident power $Y/\sqrt{P}$, as a function of the logarithm of the power, since:

$$\frac{Y}{\sqrt{P}} \propto \frac{1}{(1 + P/P_{1/2})^{b/2}} \tag{1}$$

where the exponent is $b = 1$ is expected for inhomogeneously broadened lines, while $b = 3$ is expected for homogeneously broadened lines. $P_{1/2}$ is a characteristic power, at which level the H_1 field in the cavity is such that the saturation parameter (s) is half of its maximum value ($H_{1/2}$):

$$s = \frac{1}{1 + \frac{1}{4}\gamma^2 H_{1/2}^2 T_1 T_2} = \frac{1}{2}. \tag{2}$$

where the electron gyromagnetic ratio is $\gamma = 1.76086 \times 10^7$ rad/s·Gauss. In Fig. S 1 we show the progressive power saturation results of the g′=4.3 and g′=2 signals on a brain tissue.

The fit to Eq. (1) returns the $P_{1/2}$ value and the b exponent. The fitting results are shown in Table SI. These values provide only an indication of the inhomogeneous nature of the line. We therefore expect that the linebroadening is an effect of g-factor distribution, rather than due to intrinsic spin-spin interaction.

Additionally, if the Q factor of the cavity and the geometrical constant of the microwave waveguide g'' are known, one may estimate the product of the spin-lattice and spin-spin relaxation times: T_1T_2. By calculating H_1 via the relation H_1=g$''\sqrt{QP}$, with the field in Gauss, and the Power in Watts, and $g'' = 0.028$ for an 9 GHz system [2], by reorganizing Eq. (2):

$$T_1 T_2 = \frac{4}{\gamma^2 g''^2 Q P_{1/2}} \tag{3}$$

we obtain $T_1T_2 = 0.943\ \mu\text{s}^2$ for the g′=4.3 band, at 12 K. The individual relaxation times can be determined by performing a pulsed EPR experiment, which is beyond the scope of this work.

Since the spectra were acquired at 6 G_{pp} of modulation amplitude and 2.002 mW of power, in the manuscript we only analyzed the g′=4.3 band, for which the modulation amplitude and incident power have been optimized.

Moreover, when the g′=4.3 signal amplitude Y is plotted as a function of the temperature, a Curie-law behavior is observed down to 12 K (Fig. S 1 **(a)** inset), confirming the paramagnetic nature of the iron signal.

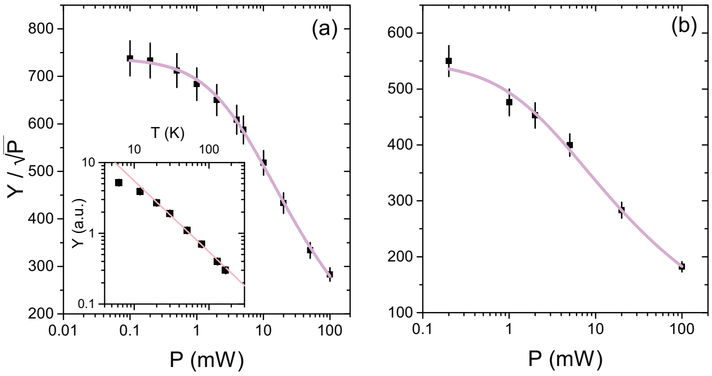

FigureS 1: Study of the relaxation times of the iron signal in brain tissue (HC). **(a)** Progressive power saturation for the band g′=4.3. The purple line is the fit to Eq. (1). Inset: Temperature dependence of the peak-to-peak amplitude of the low field signal. The pink line is a fit to the Curie law. The deviation at lower temperature may be due to line saturation [3]. **(b)** Progressive power saturation for the band g′=2. Measurements done at 12 K, and 6 G_{pp} of modulation amplitude.

Table S I: Characteristic power $P_{1/2}$ and exponent b, for the iron and copper signal, as derived by fitting the data in Fig. S 1 to Eq. (1).

**g′**	$\mathbf{P_{1/2}}$ **(mW)**	**b**
4.3	4.36 ± 0.45	0.62 ± 0.02
2	2.2 ± 0.70	0.6 ± 0.04

SIMULATION OF COPPER BAND

Hereafter we describe the Hamitonian parameters used for the Copper signal simulation (Fig. S 2). The g′=2 signal can be simulated by type-II Cu, which is described by the Hamiltonian:

$$H_{Cu} = g\mu_B(\mathbf{B} \cdot \mathbf{S}) + \mathbf{S} \cdot \mathbf{A} \cdot \mathbf{I}, \tag{4}$$

where **A** is the Hyperfine interaction with Cu nuclear spins [4]. We note that the Cu signal is overmodulated and saturated at the working point of 20 dB and 6 G_{pp}, therefore these parameters have to be considered as an approximation.

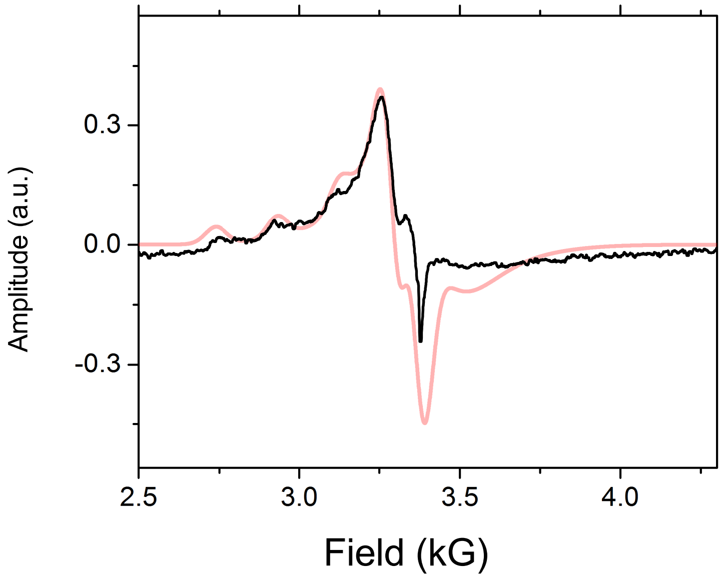

FigureS 2: Raw EPR data of the g′=2 region measured for the HC sample. The black line is the data, while the pink line is the simulation with the parameter showed in Table S II.

Table S II: Hamiltonian parameters of the human brain sample (HC). For the simulation of the copper spectrum, a second isotropic line centered at g′=2, approximately 320 G broad, was added.

***Cu* type**	**A_z (G)**	**g_x**	**g_y**	**g_z**
II	210	2.059	2.059	2.24

CONSIDERATIONS ON THE BASELINE CORRECTION OF THE EPR SPECTRA

Recently, Fadi *et al.* showed that by using a solution of Fe(III)-EDTA as a standard, 100% of the transferrin-bound iron can be estimated by the second integral of the g′=4.3 signal, with an error of 5%. When the sample contains a small (i.e. sub mM) spin concentration, as in the case of tissue, a flat baseline is a prerequisite for accuracy. Conventionally, a

polynomial baseline correction is performed on the raw data, as well as on the first integral. However, this second step may substantially affect the second integral value, and introduce more noise. In order to overcome this problem, we took the second integral of the raw data simulation, which has no baseline, by definition. The second integral is then compared to the standard iron sample.

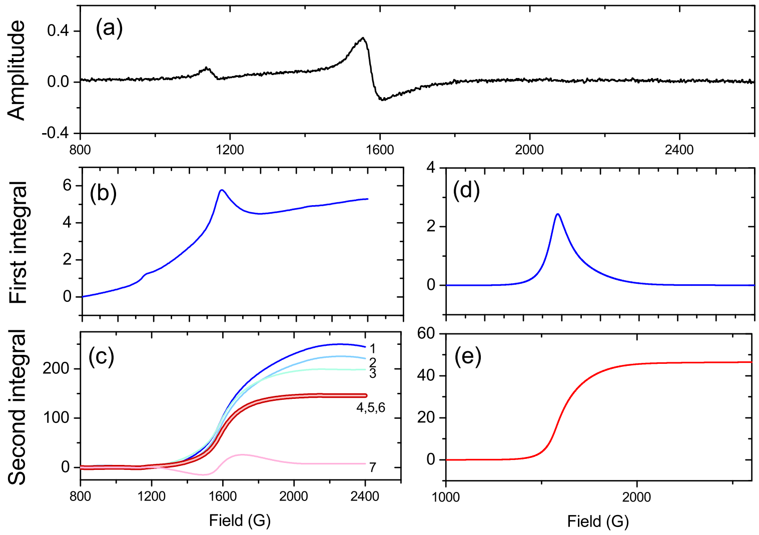

FigureS 3: 9 GHz EPR data of the tissue of AD patient, and second integral analysis. Raw EPR data, at 12 K **(a)**, over the range 800-2600 G. **(b)** First integral of the EPR spectrum, showing a large background, that has to be subtracted in order to derive the correct spin number. **(c)** Second integral, after subtracting polynomial fits of different orders (1-7 as indicates). **(d)** First integral of the simulated spectrum (which intrinsically has no background). **(e)** Second integral of the simulated spectrum.

Fig. S 3 shows how different polynomial fits of the background of the first integral may result in a spread of second integral values, thus affecting the accuracy of the analysis. It is therefore crucial, when performing quantitative EPR of highly diluted spins, to have an accurate knowledge of the Hamiltonian parameters.

For the simulations of the Fe(III) spectra, different models were tested, in order to take into account the line broadening. The best results were found by introducing the gStrain parameter, in the Easyspin package. gStrain defines the g spread for the electron spin. The distributions are assumed to be completely uncorrelated. The best fit was obtain by taking gStrain equal to $[0.52, 0.013, 0.025]$, for the Fe spectrum, and $[0.045, 0.045, 0.055]$ for Cu type-II.

[1] A. M. Portis, Phys. Rev. **91**, 1071 (1953).

[2] M. Sahlin, A. Gräslund, and A. Ehrenberg, Journ of Magn. Res. **67**, 135 (1969).

[3] C. P. J. Poole, *Electron Spin Resonance*, edited by J. Wiley and Sons (Wiley-Interscience, 1983).

[4] M. Fittipaldi, H. J. Wijma, M. P. Verbeet, G. W. Canters, and M. Huber, Applied Magnetic Resonance **30**, 417 (2006).
